# Investigation of Dispersion Kinetics of Particulate Lubricants and their Effect on the Mechanical Strength of MCC Tablets

**DOI:** 10.1007/s11095-023-03602-0

**Published:** 2023-09-26

**Authors:** Daniel Puckhaber, Arno Kwade, Jan Henrik Finke

**Affiliations:** 1 Institute for Particle Technology, Technische Universität Braunschweig, Braunschweig, Germany; 2 Center of Pharmaceutical Engineering (PVZ), Technische Universität Braunschweig, Braunschweig, Germany

**Keywords:** dispersion kinetics, lubrication, tableting

## Abstract

**Introduction:**

Tablets are commonly produced by internally adding particulate lubricants, which are known to possibly lower the mechanical strength of tablets. This reduction is caused by the coverage of matrix forming components by lubricant particles, resulting in decreased interparticulate interactions. The known incompatibilities with some active compounds of the predominantly used lubricant, magnesium stearate, call for the in-depth characterization of alternative lubricants.

**Purpose:**

Investigation of the dispersion behavior of five commonly applied pharmaceutical lubricants by mathematically modeling the dispersion kinetics for short and extended mixing times.

**Methods:**

The dispersion behavior of five different pharmaceutical lubricants were examined by systematically varying lubricant concentration and mixing time of binary formulations and evaluating the kinetic of tensile strength reduction by theoretically estimating the surface coverage based on particle sizes.

**Results:**

For short mixing times, a unifying relationship between compactibility reduction and theoretical surface coverage was identified. Subsequently, for extended mixing times, distinct differences in the shear strength and dispersion kinetics of the investigated lubricants were found.

**Conclusions:**

The lubricant particle size controls the tensile strength reduction if short mixing times are applied. For extended mixing times, the investigated lubricants can be divided into two groups in terms of dispersion kinetics. Possible underlying reasons are discussed in detail in order to enhance the general understanding of lubricant dispersions in tablet formulations.

**Supplementary Information:**

The online version contains supplementary material available at 10.1007/s11095-023-03602-0.

## Introduction

Lubricants are amongst the most commonly applied excipients in tablet formulations, as they facilitate the tablet ejection after the compaction process [1]. Their addition reduces the risk of tooling wear and some tablet defects by decreasing the friction between inner die wall and lateral tablet surface. Typically, particulate lubricants are used in pharmaceutical tablet formulations. The most commonly applied lubricant is magnesium stearate (MgSt) [2] which explains its prominence in scientific literature. Accordingly, the majority of literature focuses on the properties of MgSt [3–6] and its effect on compaction and tablet quality attributes [7–10].

Similar to most pharmaceutical lubricants, MgSt’s acting mechanism is assumed to be boundary lubrication [11, 12]. Therefore, lubricant particles adhere onto the powders they are admixed to [13]. The adhered lubricant particles form a layer at the boundary between inner die wall and tablet and by that, fill asperities at the interface [14]. Thus, the shear strength of the formed junction is lowered and the necessary force to eject the tablet from the die is reduced [12]. Conversely, the dispersion of lubricant particles onto the surface of particles within the formulation is known to affect critical tablet quality attributes [7, 15–18]. Shah and Mlodozeniec hypothesized that the mechanism of lubricant dispersion of MgSt can be divided into two stages [13]. First, lubricant particles adhere to the surfaces of other particles in the blend. Secondly, as further shear stress is applied, still-existing agglomerates of lubricant particles deagglomerate and individual particles delaminate into platelets, which then cover additional particle surfaces. The resulting coverage by lubricant particles is accompanied by a prolonged disintegration time [7, 13, 19] due to their hydrophobic nature and can severely affect mechanical strength due to the reduction of interparticulate interactions [15, 18, 19]. According to the model of Rumpf, the tensile strength of a compact is a function of number and strength of bonds formed in the agglomerate [20]. The compact porosity, the coordination number, the particle surface area and the bonding force are commonly used to describe and calculate these. In the case of lubricated powders, it is assumed that the detrimental effect on the mechanical strength can be explained by two different mechanisms [21]. The surface coverage by lubricants represents [1] a physical barrier, which prevents strong bonding between the matrix forming particles and replaces them by comparably weak bonding strengths between lubricant and matrix particles. Additionally, the [2] interparticulate distance of matrix particles may increase based on a hypothetical placeholder function of lubricant particles, decreasing effective particle interaction areas.

Furthermore, the degree of strength reduction depends on the deformation behavior of the matrix forming diluent particles [17, 18], the applied grade of MgSt [22], the applied shear stress during blending [15, 23] and a possible paddle feeder passage [16, 24, 25]. This correlation between tablet properties and dispersion behavior of MgSt is well known for decades and has been investigated in great detail. However, MgSt is known to exhibit chemical incompatibilities with some APIs [26, 27] and thereby, has to be occasionally replaced by alternative lubricants.

## Alternative Lubricants in Industrial Pharmaceutical Tablet Formulations

Generally, due to the well-known possible deterioration of tablet properties due to MgSt addition, several studies have focused on the lubrication properties of different classes of lubricants. Besides MgSt, stearic acid [18, 28, 29], glyceryl dibehenate [30–32], hydrogenated vegetable oils [33, 34], sodium stearyl fumarate [29, 31, 35], poloxamers [36, 37] and inorganic materials, e.g. talc [28, 38], were investigated as potential lubricants. Generally, most conducted studies compared only a limited number of lubricants and comprehensive studies are rare. Recently, de Backere *et al.* comprehensively investigated the impact of alternative lubricants on the tablet properties (ejection force, tensile strength and disintegration time) of three different diluents for a wide range of concentrations [37].

Most of the publications share the fact that, for the investigated alternative lubricants, a lower deterioration of the mechanical tablet strength is found at the same lubricant concentration when compared to MgSt. Qualitative explanations are typically used to explain this difference, making it challenging to transfer the results found to novel formulations. Although it is commonly believed that the acting mechanism (coverage of particle surfaces by lubricant particles as a function of applied shear stress) of boundary lubricants should be similar, quantitative models that depict the dispersion kinetics of alternative lubricants are still missing.

In order to close this gap, this study focuses on the dispersion kinetics of five different lubricants (MgSt, sodium stearyl fumarate, stearic acid, glyceryl dibehenate, hydrogenated vegetable oil) on a model material. Thus, a previously established methodology to model the impact of lubrication on the compactibility was used to develop a unifying relationship between the reduction of mechanical strength and applied lubricant concentration for the different classes of investigated lubricants. Therefore, the particle size-based theoretical surface coverage has been estimated based on easily measurable particle properties. Secondly, for a given theoretical surface coverage, the lubricant dispersion kinetics were investigated and substantial differences of the observed shear strength and dispersion kinetics between the different lubricant classes were found. By applying the presented approach, a general methodology to assess the dispersion kinetics of lubricants is presented, which can be a valuable tool for tablet formulation development.

## Materials and Methods

### Materials

Microcrystalline cellulose (MCC; Vivapur 200®, JRS Pharma GmbH, Germany) was used as diluent. Magnesium stearate (MgSt; Ligamed MF-2-V, Peter Greven GmbH, Germany), sodium stearyl fumarate (SSF, PRUV®, JRS Pharma GmbH, Germany), stearic acid (SA; Ligamed SA-1-V, Peter Greven GmbH), hydrogenated vegetable oil (HVO; LUBRITAB®, JRS Pharma GmbH, Germany), and glyceryl dibehenate (GDB; COMPRITOL® 888 ATO, Gattefossé, France) were used as lubricants. Lubricants were deagglomerated by means of a 355 μm sieve before being mixed.

### Scanning Electron Microscopy

Particle morphology was investigated by means of scanning electron microscopy (Helios G4 CX, Thermo Fisher Scientific, USA). All samples were sputtered with gold (LEICA EM ACE600, Leica microsystems GmbH, Germany) prior to measurements.

### Solids Density

Solids densities *ρ*_*s*_ were measured by means of helium pycnometry (Ultrapyc 1200e, micromeritics, USA). For each material, three samples were investigated in a ten-fold measurement and an average solids density was calculated.

### Particle Size Distribution

Particle size distributions of investigated materials were measured by means of laser diffraction (MasterSizer 3000, Malvern Panalytical, United Kingdom), applying the Fraunhofer diffraction theory. Powders were dispersed by using the dry dispersion unit AERO S and applying a dispersion pressure of 0.5 bar (Figure 2). By taking into account the solids density *ρ*_*s*_ the mass specific surface area *S*_*LD*_ was calculated by the software (Table I). Three samples of each powder were measured and an average particle size distribution was calculated.

**Table I:** Bulk Properties of Lubricants

Lubricant	x_50_ [μm]	S_LD_ [m^2^ g^-1^]
MgSt	10.2	1.092
SSF	10.9	1.151
SA	53.3	0.319
HVO	92.3	0.095
GDB	55.1	0.168

### Blending

Binary blends of 50 g consisting of MCC and the different lubricants (0.25 – 5 wt.%) were produced by means of a Turbula mixer (T2F, Willy A. Bachofen AG, Switzerland) using 1 L glass bottles. In case of MgSt, the lubricant weight fraction was limited to 4 wt.%, as higher concentrations resulted in capping of tablets. The impact of lubricant concentration on the mechanical tablet strength was quantified by applying a constant mixing time of 2 min and a blender rotation frequency of 49 min^-1^. In order to investigate the lubricant dispersion kinetics during mixing, the mixing time was varied between 2 – 120 min at a blender rotation frequency of 49 min^-1^. Here, the lubricant weight fraction *ξ*_*lub*_ was calculated acc. to the equations (4 – 8) while aiming at a particle size-based theoretical surface coverage of 2.7 % (Table II).

**Table II:** Applied Lubricant Weight Fraction to Achieve a Particle Size-Based Theoretical Surface Coverage of Approx. 2.7 %

Lubricant	*ξ*_*lub*_ for *SC*_*theo*_ ≈ 0.027
MgSt	0.005
SSF	0.006
HVO	0.04
SA	0.023
GDB	0.024

### Compaction

Formulations were compacted by a compaction simulator (Styl’One evolution, Medelpharm, France) equipped with 11.28 mm flat-faced round tooling. Dies were filled by using a gravity feeder to obtain a tablet weight of approx. 450 mg. The compression profile of a camshaft (Styl’Cam, Medelpharm, France) at a simulated rotation frequency of 5 min^-1^ was applied. This compression profile setting corresponds to a maximum punch velocity during compaction of ~ 20 mm s^-1^ and a dwell time (calculated as time period in which 90% of the maximal force is exerted) of ~ 150 ms. Axial compaction stress was systematically varied between 50 – 300 MPa. For each set of parameter and formulation, five tablets were produced and stored over night at lab conditions (20 ± 2 °C, 40 ± 10 % r.H.).

### Tablet Characterization

Tablet weight *m*_*t*_ was measured. The tablet diameter *d*_*t*_ and tablet height *h*_*t*_ was obtained by means of a tablet tester (MultiTest 50 FT, Dr. Schleuniger, Switzerland). By taking into account the solids density *ρ*_*s*_, the tablet porosity *ε* was calculated:

1 $$\varepsilon =1-\frac{4 \times {m}_t}{\pi \times {d}_t^2\times {h}_t\times{\rho}_s}$$

Subsequently, a diametric compression test at a loading rate of 0.35 mm s^-1^ was performed and the necessary breaking force *F*_*B*_ was recorded. The tablet tensile strength was calculated according to the equation of Fell and Newton [39]:

2 $${\sigma}_t=\frac{2\times {F}_B}{\pi \times {d}_t\times {h}_t}$$

## Results and Discussion

### Correlation of Compactibility Reduction and Theoretical Surface Coverage for Short Mixing Times

**Fig. 1:**
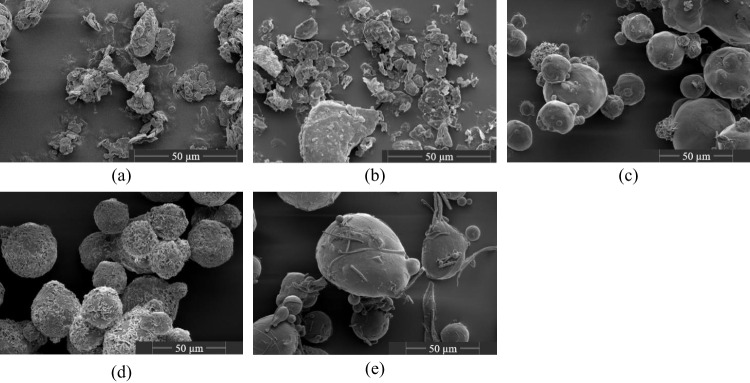
SEM images of lubricants. (**a**) MgSt, (**b**) SSF, (**c**) SA, (**d**) HVO, (**e**) GDB.

**Fig. 2:**
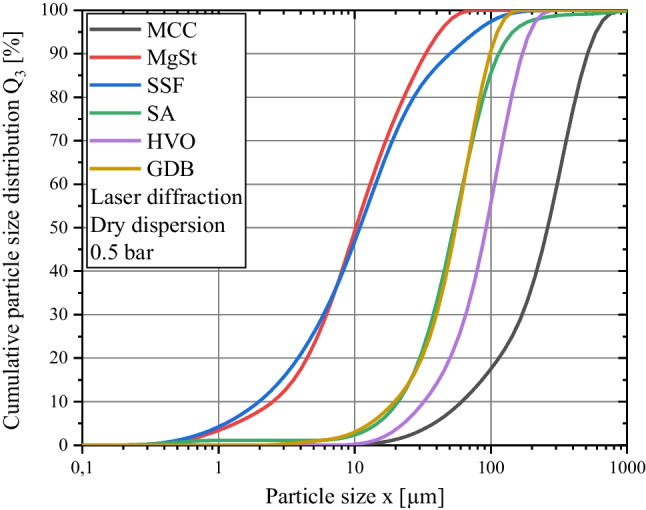
Cumulative particle size distribution of investigated excipients.

**Fig. 3:**
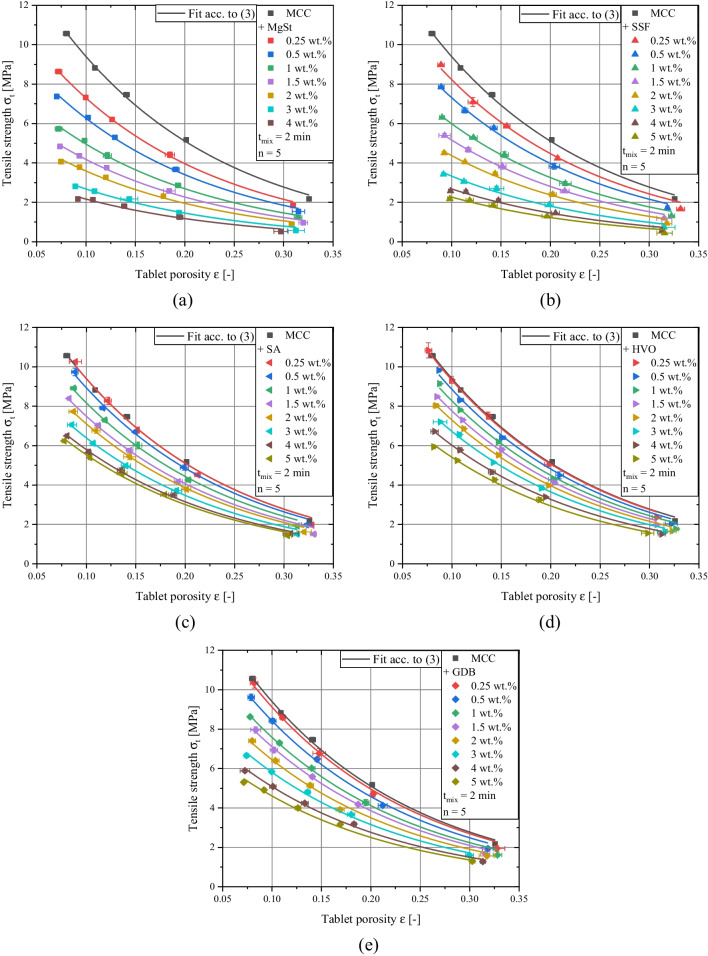
Compactibility profiles of binary mixtures of MCC + (**a**) MgSt, (**b**) SSF, (**c**) SA, (**d**) HVO, (**e**) GDB for varying lubricant concentrations for a fixed mixing time of 2 min. Lines represents fit acc. to Eq. (3) whereby the bonding capacity of MCC (*k*_*b*_ = 6.1) was applied. Plotted data represent mean values and standard deviations, respectively.

The particle morphology of the investigated lubricants and the cumulative particle size distributions of the applied materials are shown in Fig. 1 and Fig. 2, respectively. The effect of lubrication on the mechanical strength can be evaluated by investigating the compactibility profiles which relate tablet porosity to tablet tensile strength [40] with rising amount of lubricant, the tablet tensile strength for a given tablet porosity is decreasing due to the increased coverage of diluent surface by lubricant particles (Fig. 3a-e). The extent to which the compactibility is affected depends on the applied lubricant. Generally, the reduction of tensile strength is particularly pronounced when using flake-like, smaller particles of MgSt and SSF and less distinct for spherical, bigger particles of SA, HVO and GDB. This qualitative finding is in good agreement with previously conducted studies which also found a higher loss of tensile strength for lubricants exhibiting a flake-like morphology [29, 37]. In order to further elucidate the compactibility reduction due to lubrication, the compactibility profiles were quantitatively evaluated.

Therefore, the compactibility profiles were mathematically modeled by the Ryshkewitch-Duckworth equation, which relates the tablet tensile strength *σ*_*t*_ to the tablet porosity *ε* by two empirical fit parameters [41, 42]:

3 $${\sigma}_t={\sigma}_0\times {e}^{-{k}_b\times \varepsilon }$$

Where *k*_*b*_ is commonly referred to as bonding capacity and *σ*_*0*_ to the theoretical maximal tensile strength which relates to the assumed tensile strength of a non-porous tablet. For the majority of pharmaceutical materials, non-porous tablets cannot be formed by compaction and thus, *σ*_*0*_ was determined by extrapolation based on the available compactibility data.

**Fig. 4:**
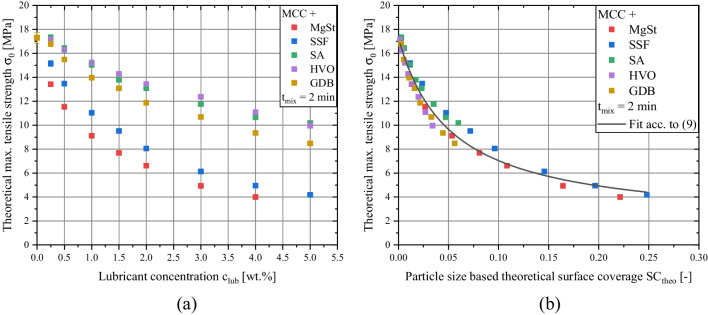
Correlation of theoretical max. tensile strength values *σ*_*0*_ of investigated formulations with lubricant concentrations (**a**) and with particle size based-theoretical surface coverage (**b**). Formulations were produced by applying a mixing time of 2 min.

In a previous study, it could be shown that the impact of lubrication on the bonding capacity of MCC is minor [40]. Thus, it was assumed that *k*_*b*_ remains constant and can be estimated by using the value derived for the pure diluent (*k*_*b,MCC*_ = 6.1). For all measured compactibility profiles, a sufficiently high correlation coefficient (> 0.97) resulted for the described method. Subsequently, the impact of the internally added lubricant is limited to the change of the theoretical maximal tensile strength *σ*_*0*_. As expected by the compactibility plots, *σ*_*0*_ decreases with rising lubricant concentrations (Fig. 4a). Additionally, it is evident that the highest compactibility reduction is found when MgSt or SSF are applied. In contrast, for SA, GDB and HVO the theoretical max. tensile strength values are considerably higher for a given lubricant concentration. These findings are generally in good accordance with previously conducted studies, which found higher mechanical tablet strength values for formulations containing alternative lubricants instead of MgSt [18, 31, 37]. Different possible underlying reasons were discussed. Shah *et al.* proposed a smaller interference of particle bonding for SSF and GDB [31] while de Backere *et al.* suggested that found deviations may be explained by differences in lubricant particle size and shape [37]. However, there is still no conclusive evidence which mechanism is responsible for the observable differences between the lubricants. Consequently, there is no quantitative correlation between the applied lubricant and mechanical strength reduction for different lubricant types available in the literature.

Microcrystalline cellulose was chosen as model material as it is known to plastically deform during compression resulting in the limited generation of new surfaces by particle fragmentation [43, 44]. Thus, it is assumed that the mixing process defines the extent to which MCC particle surfaces are covered by lubricant particles and consequently, determines the reduction of interparticulate interactions [7, 13, 18]. Hence, the coverage by lubricant particles has to be taken into account. The experimental measurement of the surface coverage is challenging due to the small lubricant amount, their small and usually irregular particle size as well as the non-spherical shape of typical diluents. Consequently, the surface coverage was theoretically estimated based on an adapted approach of Meyer [45]. This approach was already successfully applied by our research group in a previous publication to estimate the theoretical surface coverage by lubricant particless for multi-component mixtures [46].

The theoretical surface coverage was calculated by assuming that the available diluent surface *S*_*dil*_ can be reasonably estimated by its mass specific surface area *S*_*LD*_ derived by laser diffraction multiplied with the total diluent mass *m*_*dil*_:

4 $${S}_{dil}={S}_{LD, dil}\times {m}_{dil}$$

**Fig. 5:**
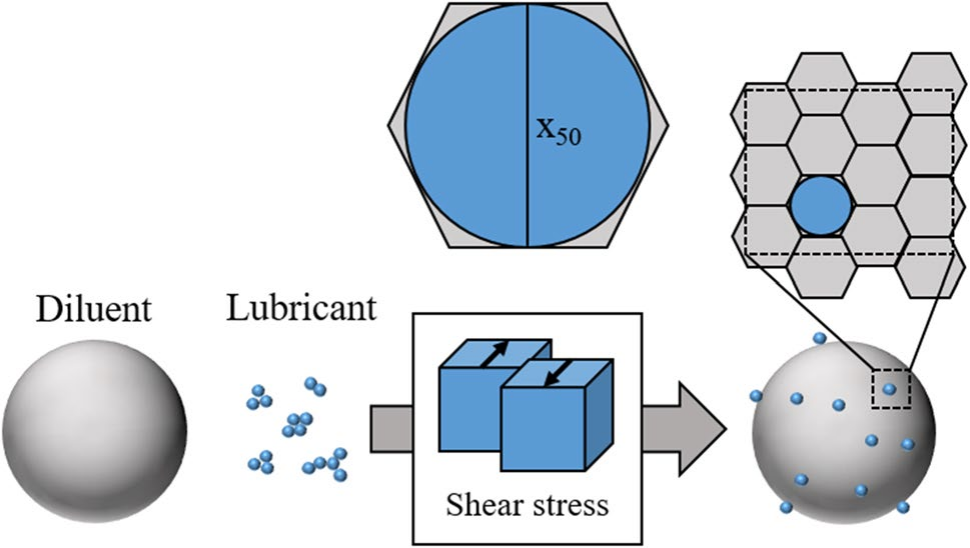
Schematic representation of particle size based theoretical surface coverage by assuming that each lubricant particle occupies the hexagonal shaped surface according to its median particle size.

The densest possible monolayer coverage by spherical guest particles is achieved, if every guest particle occupies the space of a hexagon *S*_*lub,particle*_ according to its median particle size *x*_*50*_ (Fig. 5) [45]:

5 $${S}_{lub, particle}=\frac{\sqrt{3}}{2}\times {x}_{50}^2$$

In order to calculate the total number of lubricant particles, the mass of a single lubricant particle *m*_*lub,particle*_ was estimated:

6 $${m}_{lub, particle}=\frac{\pi }{6}\times {x}_{50}^3\times {\rho}_s$$

The total occupied surface by lubricant particles can then be yielded by:

7 $${S}_{lub}={S}_{lub, particle}\times {n}_{lub}={S}_{lub, particle}\times \frac{m_{lub}}{m_{lub, particle}}$$

Where *n*_*lub*_ is the total number of lubricant particles and *m*_*lub*_ is the total lubricant mass. Relating the available and occupied surface yields the particle size-based theoretical surface coverage *SC*_*theo*_:

8 $$S{C}_{theo}=\frac{S_{lub}}{S_{dil}}$$

In the scope of this approach, it was assumed that the lubricant particles exhibit a spherical shape and an uniform particle size. As evident by SEM images (Fig. 1) and particle size distributions (Fig. 2), these assumptions are not completely valid. However, there is currently no easily applicable method available to consider the distributed particle size and particle shape on the theoretical surface coverage. Thus, the median particle size *x*_*50*_ derived by laser diffraction was used as a characteristic particle size value. Additionally, it has to be noted that the particle size distributions of lubricants strongly depend on the applied dispersion stress during particle size measurement. This is especially valid for MgSt, which is believed to delaminate into smaller particles due to acting shear stresses [13]. In order to limit this effect and capture the particle size during the beginning of the blending process, a minimal air pressure of 0.5 bar was applied for the particle size analysis.

**Fig. 6:**
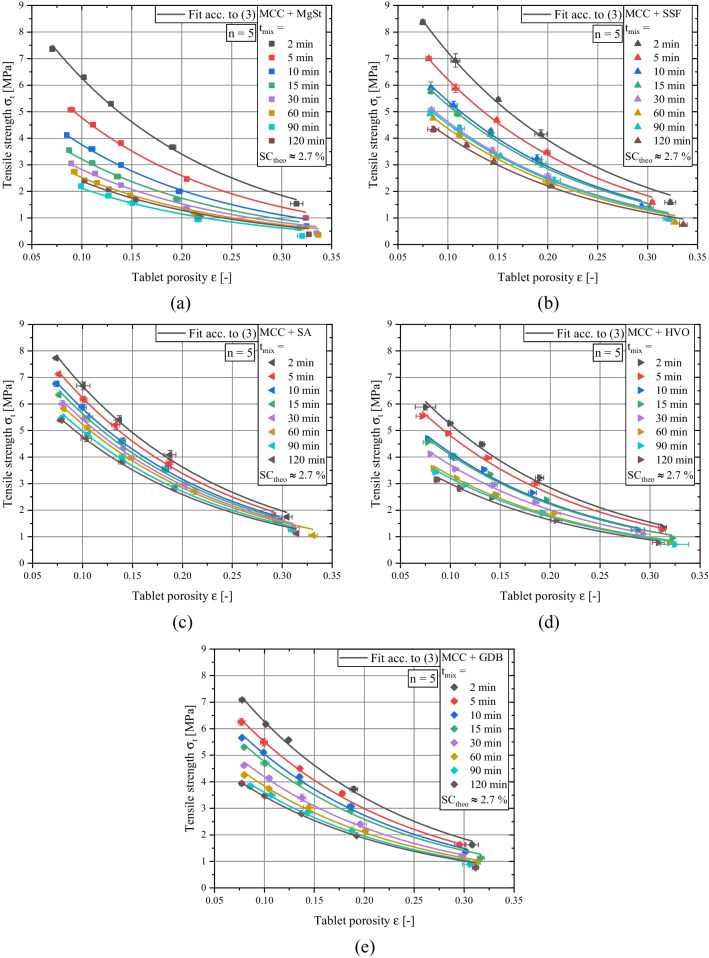
Compactibility profiles of binary mixtures of MCC + MgSt (**a**), SSF (**b**), SA (**c**), HVO (**d**) and GDB (**e**) for a fixed initial particle size-based theoretical surface coverage of ≈ 2.7 % for different mixing times. Lines represent fits acc. to Eq. (3) whereby the bonding capacity of MCC (*k*_*b*_ = 6.1) was applied. Plotted data represent mean values and standard deviations, respectively.

**Fig. 7:**
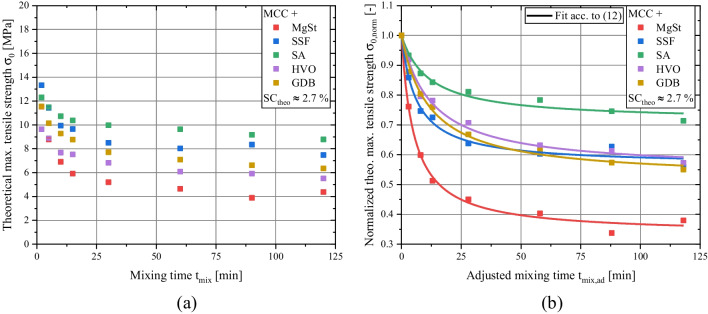
Dispersion kinetics of binary mixtures of MCC and lubricants for a *SC*_*theo*_ value of approx. 2.7 % for different mixing times. Absolute values of *σ*_*0*_ (a) and normalized values of *σ*_*0,norm*_ (b).

**Fig. 8:**
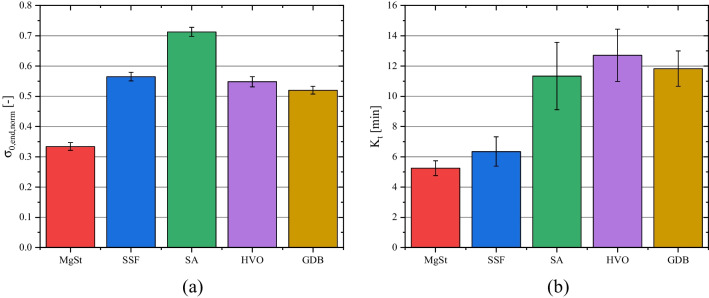
Derived *σ*_*0,end,norm*_ and *K*_*t*_ values of the dispersion kinetics of investigated lubricants for a particle-based theoretical surface coverage of approx. 2.7 % in a blend with MCC, while varying the mixing time between 2 and 120 min. Error bars represent standard deviation derived by fitting data with Eq. (12).

**Fig. 9:**
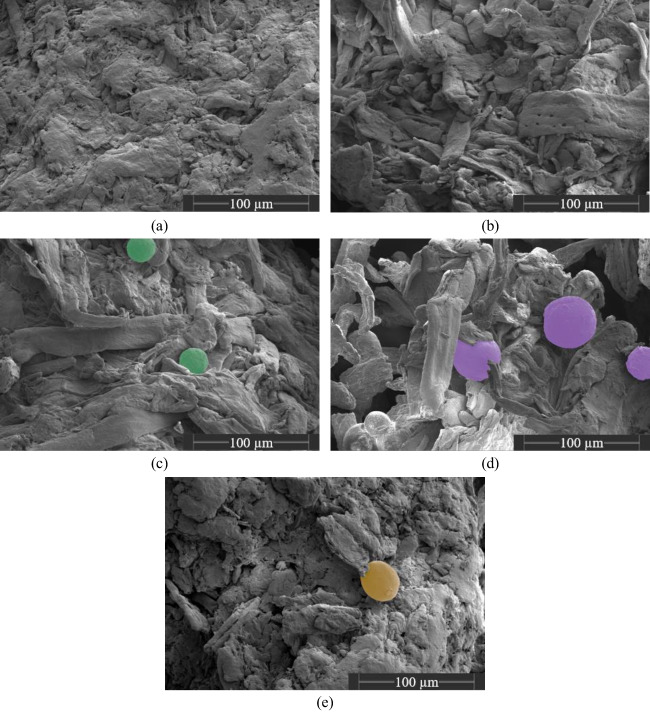
Representative SEM images of the outer surface of MCC particles for binary formulations of MCC + MgSt (**a**), + SSF (**b**), + SA (**c**), + HVO (**d**) + GDB (**e**). Formulations were produced for a *SC*_*theo*_ value of approx. 2.7 % and mixed for 120 min. Visually detectable SA, HVO and GDB particles were colored for interpretation purposes.

Consequently, *σ*_*0*_ values were plotted against the particle size-based theoretical surface coverage and revealed a unifying relationship for the investigated binary formulations (Fig. 4b). This compactibility reduction was modeled by an adapted dispersion kinetic proposed by Schilde *et al.* [47] while parameters were modified, accordingly:

9 $${\sigma}_0={\sigma}_{0, dil}+\left({\sigma}_{0, end}-{\sigma}_{0, dil}\right)\times \frac{SC_{theo}}{SC_{theo}+{K}_{SC}}$$

Where *σ*_*0,dil*_ is the *σ*_*0*_ value of the unlubricated diluent, *σ*_*0,end*_ is the minimal *σ*_*0*_ value at full dispersion and *K*_*SC*_ is a kinetic constant. Fitting the data by Eq. (9) results in a high correlation coefficient (R^2^ > 0.96). Alternative dispersion kinetics [23, 24] were also considered, but yielded poorer correlation.

No plateau was reached for the investigated range of *SC*_*theo*_, thus, the *σ*_*0*_ value at full dispersion (*σ*_*0,end*_) is extrapolated. However, as the applied lubricant concentrations are well within the industrially relevant concentration range, the endpoint is only of theoretical relevance. Knowledge of this relationship allows the prediction of the expected compactibility reduction for a chosen lubricant concentration with high accuracy. The necessary information to conduct this prediction are easily assessable, which highlights the value of the identified relationship between lubricant particle size and compactibility reduction. Remaining visible discrepancies of this relationship for certain lubricants are small (max. absolute error of ~ 1.25 MPa) and probably caused by the different deviation of the actual from the assumed spherical particle shape, differences in the particle size distribution width which cannot be described by *x*_*50*_ values, and minor differences in the dispersion behavior. Additionally, the prediction strength of the model is investigated by predicting the tensile strength and comparing it to the experimentally determined values (Supplementary 1). Here, the prediction is carried out by using the parametrized fit function (Fig. 4b) to determine *σ*_*0*_ and subsequently applying Eq. (3) to calculate the predicted tensile strength. For all investigated formulations, a high prediction accuracy was obtained, as expected by the data shown in Fig. 4b.

Consequently, Fig. 4b clearly shows that the mechanical strength reduction of tablets due to lubrication is mainly governed by the characteristic lubricant particle size, represented by the particle size-based theoretical surface coverage, when short mixing times are applied. This finding can be seen as an extension of the previously found linear relationship between tensile strength and mean MgSt particle diameter [9] for a larger range of investigated particle sizes and additional validity for different lubricant types. Additionally, by taking into account the mechanism of effect (coverage of diluent surface by lubricant particles), the proposed methodology also incorporates the available diluent surface and should be generally transferable to different diluent particle sizes. Thus, a simple and easily applicable method to predict the loss of mechanical strength due to lubrication, which was proven valid for different chemical lubricant entities, was developed.

However, this correlation and its transferability to alternative process parameters and diluents must be critically evaluated. As shown in several studies, prolonged mixing times result in further deterioration of the mechanical strength of tablets due to the ongoing dispersion of lubricant particles [7, 13, 15, 19]. As discussed later, this dispersion behavior heavily depends on the applied lubricant. Thus, the applied short mixing time of 2 min represents only an estimation of the mixing time where lubricant agglomerates are dispersed but no further dispersion of individual lubricant particles take place. Furthermore, the particle sized-based theoretical surface coverage might not necessarily represent the real surface coverage but the good to very good description of the measured values by the model proves the quality of this approach. This method was currently only evaluated using the model material MCC. If no specific interaction between the diluent and lubricant particles exists and the applied diluent shows a measurable sensitivity to lose mechanical strength due to reduced bondability, this methodology should also generally be applicable to other diluent materials. Nonetheless, this has to be evaluated in future studies.

### Dispersion Kinetics of Applied Lubricants for Prolonged Mixing Times

The lubricant dispersion kinetics for prolonged mixing times were examined by evaluating the compactibility reduction as a function of mixing time. Therefore, binary mixtures were produced by a 3D-shaker mixer while systematically varying the mixing time between 2 – 120 min. As shown in the previous section, for short mixing times, the compactibility reduction is mainly governed by the median lubricant particle size. In literature, it is assumed that lubricant particles are initially deposited onto diluent surfaces and thereafter, acting shear stress results in a further particle dispersion. This particle dispersion at prolonged mixing times was explained by delamination of individual lubricant particles into smaller particles which are subsequently deposited on previously uncovered diluent surfaces [13]. Generally, previously conducted studies mainly focused on the dispersion behavior of MgSt, as it is the predominantly applied pharmaceutical lubricant [7, 13, 15, 17, 48]. Comprehensive comparative studies of dispersion behavior as a function of mixing time for various pharmaceutically relevant lubricants are not available, yet.

In order to quantify the lubricant dispersion for prolonged mixing times, a common particle size-based theoretical surface coverage *SC*_*theo*_ of ≈ 2.7 % was applied. This value was chosen according to the resulting *SC*_*theo*_ value for a MgSt concentration of 0.5 wt.% and calculated by equations (4 – 8). The resulting weight fractions of the investigated lubricants are enlisted in Table II. Consequently, comparable compactibility profiles for a mixing time of 2 min should result. Afterwards, the compactibility reduction as a function of mixing time was evaluated. Depending on the applied lubricant, the compactibility reduction due to the prolonged mixing time considerably varies (Fig. 6a-e). In order to quantify the extent of compactibility reduction, the derived compactibility profiles were fitted by Eq. (3) while keeping the bonding capacity constant (*k*_*b,MCC*_ = 6.1). Thereby, the impact of mixing time on reduction of mechanical strength can be directly correlated to the change of the theoretical max. tensile strength *σ*_*0*_.

Plotting the *σ*_*0*_ values against the mixing time allows the evaluation of the dispersion behavior of the applied lubricants (Fig. 7a). It is evident that for all investigated lubricants, a reduction of mechanical strength for higher mixing times is observed. Additionally, the *σ*_*0*_ values approach a threshold that corresponds to the maximum lubricant dispersion for the given experimental setup. Here, for the highest mixing times, the smallest *σ*_*0*_ values were found for MgSt in contrast to the investigated alternative lubricants. This finding is in good accordance with previously conducted studies, which found a considerable decrease of tensile strength of tablets containing MgSt for prolonged mixing times [15, 19].

However, it is also visible that despite the application of a common *SC*_*theo*_ value, the *σ*_*0*_ values for a mixing time of 2 min vary. Those differences can be explained by different ambient conditions (relative humidity), random errors (weighing and measurement inaccuracies) and already discussed small deviations of the previously identified unifying relationship (Fig. 4b). Therefore, in order to enhance the comparability between the investigated formulations, the normalized *σ*_*0*_ values were calculated by relating the *σ*_*0*_ values for prolonged mixing times to the *σ*_*0*_ values determined for a mixing time of 2 min:

10 $${\sigma}_{0,\mathit{\operatorname{norm}}}=\frac{\sigma_{0,t}}{\sigma_{0,2\ \mathit{\min}}}$$

In principle, it is debatable if a mixing time of 2 min leads to a state where the lubricant distribution depends only on the median lubricant particle size and not on additional dispersion mechanisms, e.g. delamination. As no further short mixing times were evaluated in the course of this study, the applied value of 2 min represents an arbitrarly chosen value which represents such short mixing times in general. Nonetheless, the previously developed unifying relationship between *σ*_*0*_ and *SC*_*theo*_ for a short mixing time of 2 min (Fig. 4b) indicates that the assumption of a comparable, minimal lubricant dispersion for the investigated mixing time of 2 min is reasonable.

Furthermore, the mixing time was adjusted in order to focus on the reduced compactibility in relation to the normalized *σ*_*0,norm*_ determined at a mixing time of 2 min:

11 $${t}_{mix, ad}={t}_{mix}-2\ \mathit{\min}$$

Plotting *σ*_*0,norm*_ against *t*_*mix,ad*_ allows the evaluation of the sensitivity of the individual lubricants to be dispersed due to acting shear stress during prolonged mixing times (Fig. 7b). This dispersion behavior was quantified by applying the adapted Eq. (9):

12 $${\sigma}_{0,\mathit{\operatorname{norm}}}=1+\left({\sigma}_{0, end,\mathit{\operatorname{norm}}}-1\right)\times \frac{t_{mix, ad}}{t_{mix, ad}+{K}_t}$$

Where *K*_*t*_ is a kinetic constant, which describes the necessary time until 50% of the absolute reduction of compactibility is reached.

Equation (12) can be used to quantify two different properties of the dispersion behavior of the applied lubricants. The normalized minimal strength value *σ*_*0,end,norm*_ corresponds to the dispersability of the lubricant, which defines the effective particle size (and, by that, number of particles) to which it can be dispersed using the given mixing setup and the acting maximum shear stresses during mixing. Small values of *σ*_*0,end,norm*_ correspond to small effective particle sizes (and, in turn, high numbers) of dispersed lubricant particles reached in the equilibrium of the process, leading to high surface coverages and low (maximal) tensile strengths. According to this plot, the investigated lubricants can be categorized into three different groups based on their *σ*_*0,end,norm*_ values (Fig. 8a).

The smallest *σ*_*0,end,norm*_ values of 0.33 were determined for MgSt, followed by SSF, HVO and GDB which exhibited comparable values varying between 0.52 – 0.57. In contrast, blends containing SA showed a significantly higher *σ*_*0,end,norm*_ value of 0.71. Consequently, MgSt shows the smallest shear strength, a finding which is in good accordance with previously conducted research which found a substantial decrease of tablet strength for prolonged mixing times if MgSt was applied as lubricant [15, 19]. It is interesting to note, that, despite their obvious differences in particle size and shape (c.f. Fig. 1), SSF, HVO and GDB seem to exhibit comparable shear strength values. In contrast, SA displays the highest shear strength and thereby, only a limited amount of delamination and deagglomeration of lubricant particles during the ongoing mixing process occurs. Until today, there is no commonly applied method available to meaningfully characterize or even predict the shear strength of individual lubricant particles during the mixing process. In order to minimize the experimental effort and to rationalize the development of new tablet formulation, it would be desirable to develop such a method, which would allow to correlate the apparent differences in dispersion behavior with easily measurable particle properties.

The half-life *K*_*t*_ can be used to describe the kinetics of the lubricant dispersion during extended mixing times and by that displays the difference in dispersion strength. Generally, the derived *K*_*t*_ values imply two different groups of lubricant with regard to their dispersion kinetic: MgSt and SSF, exhibiting *K*_*t*_ values between 5.25 – 6.35 min and SA, HVO and GDB, whose K_t_ values ranged between 11.34 – 12.71 min. This disparity implies considerable differences in dispersion kinetics due to the efficiency of stress events to disperse the original lubricant particles into smaller particles. Two different scenarios appear as reasonable explanations:

1. The different kinetics could arise due to different dispersion mechanisms. For flake-like particles, like MgSt and SSF, it assumed that individual particle layers delaminate due to the acting shear stress. The delaminated particle layers subsequently cover the diluent surface. Consequently, individual lubricant particles cannot be identified on the diluent surface for long mixing times (Fig. 9a-b). In contrast, the spherical lubricants (SA, HVO and GDB) exhibit a very different and dense particle structure (Fig. 1). Those dense structures are still visible after 120 min of mixing (Fig. 9c-e). Comparing the SEM images of the lubricant powder with the mixtures containing spherical lubricants (c.f. Figs. 1 and 9), a limited amount of deagglomeration of secondary, smaller particles seems to have occurred. However, the small number of investigated particles prevents a statistically proven statement. Additionally, due to their larger particle size, it could be hypothesized that the larger, close to spherical particles roll over the diluent surfaces during mixing and, by that, create a thin lubricant layer by smearing.
2. The differences in kinetic behavior, expressed by differences in *K*_*t*_, could arise due to differences in the number of lubricant particles at the start of the extended mixing times. The weight fraction of lubricants was selected in order to achieve a comparable particle size-based theoretical surface coverage at the start of the dispersion experiments. However, due to the differences in particle size and solid densities, constant *SC*_*theo*_ values do not correlate with a constant number of lubricants particles (Table III). It is evident that for smaller median particle sizes, the number of lubricant particles in the formulation increases. Assuming that the dispersion of lubricant (independent of the underlying mechanism) occurs only due to the acting shear stress between two diluent particles (effectively acting similar to grinding media), a higher number of lubricant particles enhances the probability that a lubricant particle is caught between two diluent particles during a stress event and, if the stress energy is sufficiently high, will be dispersed. Additionally, in absolute terms, the necessary energy for particle dispersion within one collision event is reduced for smaller particles. Consequently, it could be hypothesized that the dispersion of smaller particles (MgSt and SSF) occurs faster, as both the number of lubricant particles is increased and the necessary stress energy per contact for a successful dispersion event is decreased.

In future studies, it would be desirable to further investigate the acting dispersion mechanisms during mixing of lubricants and to correlate those mechanisms to particulate properties of the investigated formulation.

**Table III:** Number of Lubricant Particles Used in the Lubricant Dispersion Experiments. Lubricant Weight Fraction was Calculated Based on a *SC*_*theo*_ Value of Approx. 2.7 %. Number of Particles was Calculated Based on the Assumption That All Lubricant Particles Exhibit a Spherical Shape, no Intraparticulate Porosity and a Constant Particle Size Represented by Their *x50*

Lubricant	Number of particles
MgSt	4.17 ∙ 10^8^
SSF	3.66 ∙ 10^8^
SA	1.5 ∙ 10^7^
HVO	4.92 ∙ 10^6^
GDB	1.4 ∙ 10^7^

Furthermore, the effect of shear rate was not considered in this study. However, higher shear rates will result in an accelerated lubricant dispersion due to the increased number of stress events per time and increased stress intensity. The latter could potentially also decrease the minimal compactibility found for sufficiently long mixing times as the stress intensity could exceed the materials shear strength at high degrees of dispersion. The verification of this hypothesis and a model-based description of it should be adressed in future studies.

As adequate measurement techniques to investigate the dispersion behavior of lubricants during mixing are not available, the stated hypotheses are based on surrogates (changes in tensile strength and not directly measured effective particles sizes) and cannot be directly be proven, yet. It can be presumed that in reality, a complex interaction between the lubricant structures, their shear strength and the occurring shear stresses is probably responsible for the measured different dispersion behavior. Nonetheless, it can be concluded that the dispersion behavior of the investigated lubricants considerably vary, both in terms of shear strength and dispersion kinetics. The identified trends could help to further rationalize the development of new tablet formulations.

Thus, this study can be seen as a starting point to further investigate the dispersion behavior of particulate lubricants in tablet formulations and should be carefully extended to more complex formulations and diluents with partially brittle deformation properties in future studies. For the latter, especially the interplay of reduced friction during the ejection and the reduced compactibility should be of special interest. Additionally, the currently presented findings should be transferred and used to enhance a mechanistic model (e.g. a combination [49] of the models of Rumpf and JKR) to further elucidate, which acting mechanisms are mainly responsible for the observed strength reduction by lubricant addition. At present, it is not readily applicable to disperse systems such as in the present study, but represents another interesting possibility to minimise the empirical effort and to improve the overall process understanding.

## Conclusion

In this study, it could be shown that the compactibility of highly ductile MCC is rapidly decreasing as a function of lubricant concentration. First, correlating the theoretical surface coverage (based on the particle sizes of diluent and lubricants) with the reduced compactibility revealed a unifying relationship for all investigated formulations. Thereby, it can be clearly shown that the initial reduction of mechanical strength due to lubrication is a function of the applied lubricant particle size. By applying the dispersion kinetic model of Schilde *et al.*, this correlation was quantified, which allows the prediction of the compactibility reduction of an unknown lubricant concentration. Secondary, the dispersion kinetics of lubricants for extended mixing times were investigated for a fixed initial theoretical surface coverage of  ≈2.7 %. Thereby, the reduced compactibility for extended mixing times was related to a common starting point at 2 min mixing time as the prediction of the adhesion force reduction as a function of the applied lubricant is not established, yet. The investigated lubricants showed great differences in the resulting equilibrium mechanical strength, measured after 120 min of mixing, which was correlated to their differences in effective particle size after dispersion. Additionally, two different dispersion behaviors were identified according to the kinetic constants of the applied dispersion models. Generally, small, flake-like structured particles (MgSt and SSF) showed a significantly faster dispersion during mixing compared to the spherical lubricants (SA, HVO, GDB). Conclusively, the obtained knowledge can be utilized to assess the risk of overlubrication, which has to be avoided during tablet manufacturing, and, by that improve process stability in a rational, science-based approach.

### Supplementary Information

Fig. 10 (PNG 29 kb)

High resolution image (TIF 30 kb)

Fig. 11 (PNG 33 kb)

High resolution image (TIF 29 kb)
