# Supplementary figures and images for: Investigation of Dispersion Kinetics of Particulate Lubricants and their Effect on the Mechanical Strength of MCC Tablets
Source: Pharm Res. 2023 Sep 26;40(10):2479–92. doi: 10.1007/s11095-023-03602-0 (PMC10661788; doi:10.1007/s11095-023-03602-0)

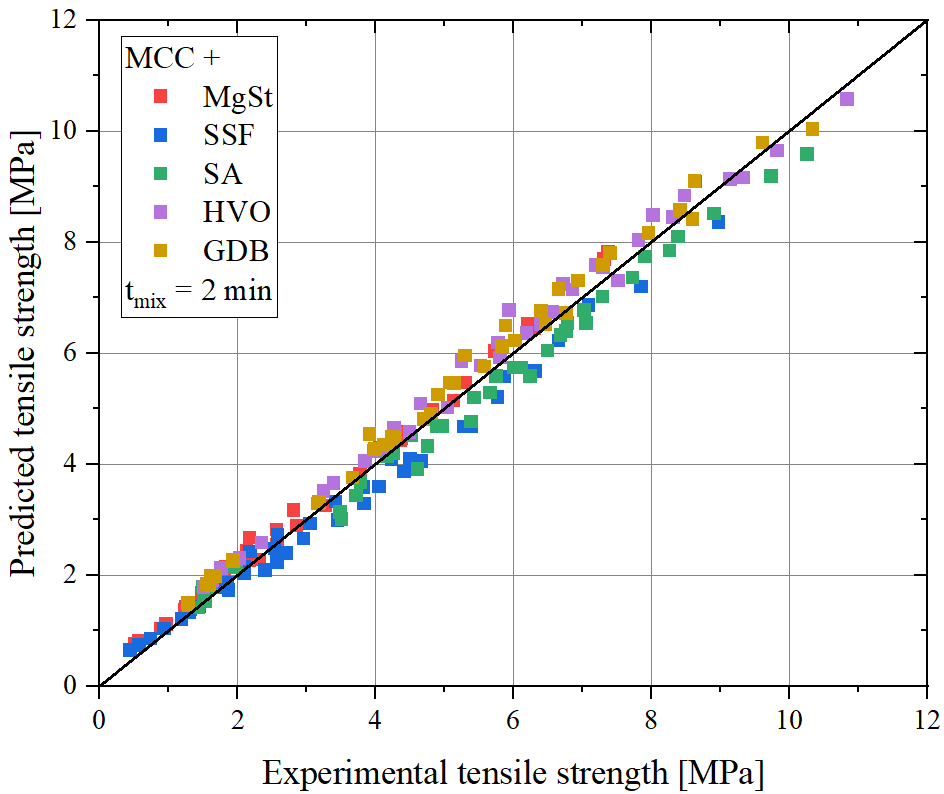

Supplement: Supplementary file 1 — (PNG 29 kb) [file 11095_2023_3602_Fig10_ESM.png]

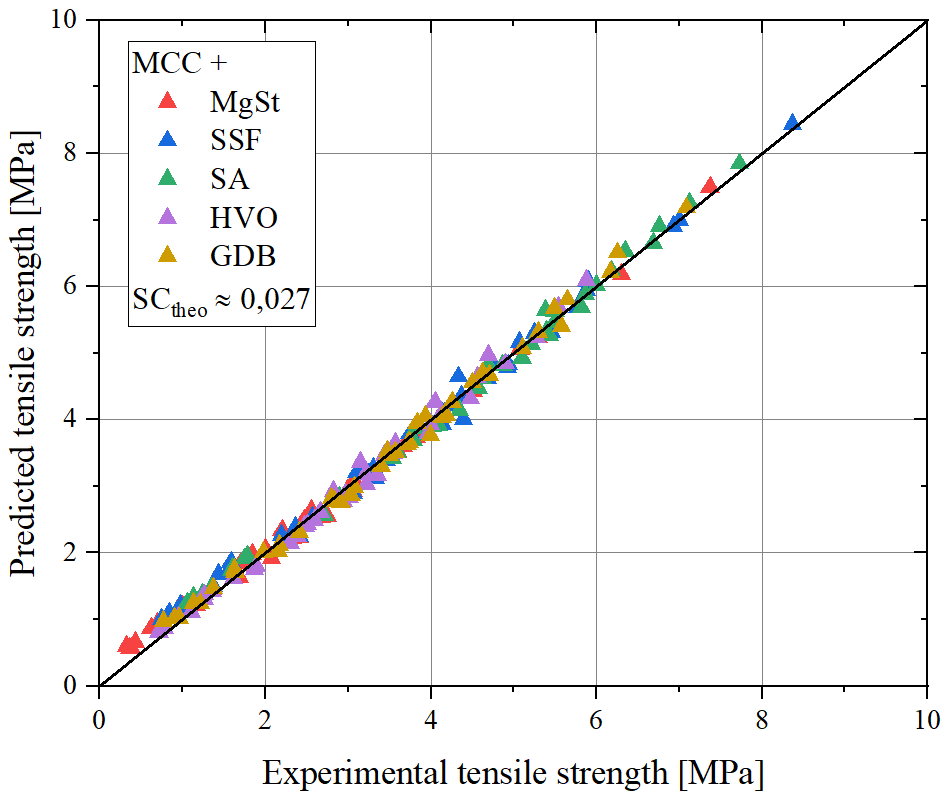

Supplement: Supplementary file 3 — (PNG 33 kb) [file 11095_2023_3602_Fig11_ESM.png]
